# Acute Whole-Body Heat Exposure Modulates EEG Microstate Dynamics, Phase-Lagged Connectivity, and Cortical Source Activity

**DOI:** 10.1007/s10548-026-01258-8

**Published:** 2026-10-07

**Authors:** Bo Chen, Li Ding, Xiaopeng Liu

**Affiliations:** 1 Air Force Medical Center, Air Force Medical University, Beijing, 100142 China; 2 School of Biological Science and Medical Engineering, Beihang University, Beijing, 100191 China

**Keywords:** Environmental temperature, EEG microstates, Functional connectivity, Source localization

## Abstract

Acute exposure to elevated ambient temperature affects physiological regulation, thermal perception, and cognitive functioning, yet its influence on the spatiotemporal organization of human brain activity remains insufficiently understood. This study investigated temperature-related changes in EEG spectral power, microstates, functional connectivity, and cortical source activity. Fifteen healthy male participants completed three randomized 45-min seated exposures at ambient temperatures of 21, 30, and 38 °C while wearing standardized winter protective clothing (3.2 clo). EEG, skin temperature, core body temperature, and subjective thermal sensation were recorded. Mean skin temperature, core body temperature, and thermal-sensation votes increased with temperature and exposure duration. Global delta-band connectivity differed significantly across temperatures (χ^2^=12.64, FDR-adjusted q=0.0090). Delta connectivity was significantly higher at 38 °C than at 21 °C. Microstate D coverage differed across temperatures, with values of 0.145, 0.178, and 0.150 at 21, 30, and 38 °C, respectively (χ^2^=12.88, FDR-adjusted q=0.0064, W=0.429). Source-space analysis further demonstrated increased relative delta source power with temperature and reduced beta and gamma source power at 38 °C, particularly in occipital, parietal, sensorimotor, and posterior cingulate regions. Increasing ambient temperature was associated with a shift in EEG spectral activity toward lower frequencies, enhanced delta-band functional synchronization, and altered microstate temporal organization. Together, these exploratory findings indicate that spectral power, regional phase-lagged connectivity, and microstate dynamics show distinct responses to ambient temperature in healthy young men wearing protective clothing.

**Supplementary Information:**

The online version contains supplementary material available at https://doi.org/10.1007/s10548-026-01258-8.

## Introduction

Acute exposure to elevated ambient temperature imposes a thermal load that can influence human physiological regulation and cognitive functioning. Exposure to heat can disrupt thermal homeostasis and affect thermal perception, alertness, attention, and behavioral performance (Hancock and Vasmatzidis 2003; Kim et al. 2020; Han and Chun 2021). Workers such as firefighters, military personnel, and hazardous-material responders may encounter high ambient temperatures while wearing highly insulating protective clothing, which can increase thermal strain. Understanding how brain activity changes under these combined conditions may help characterize the neurophysiological burden of such exposures. Because the brain is responsible for integrating thermal signals and coordinating perceptual, behavioral, and autonomic responses, characterizing temperature-induced changes in brain activity is crucial for understanding the neural basis of human adaptation to thermal environments (Hocking et al. 2001; Lv et al. 2017). However, the spatiotemporal mechanisms through which environmental temperature modulates large-scale brain activity remain insufficiently understood (Pourghorban et al. 2024).

Previous EEG research provides evidence that thermal exposure changes cortical oscillations, but the reported pattern depends on stimulus type, exposure intensity, and behavioral context (Son and Chun 2018; Niu et al. 2022). During brief non-painful warming and cooling of the hand, Chang et al. observed condition-specific changes in theta and alpha power and in interelectrode coherence (Chang et al. 2005). In indoor climate-chamber experiments, relative power in the delta, theta, alpha, and beta bands has been associated with thermal sensation, thermal comfort, and neurobehavioral performance across neutral-to-hot conditions (Lang et al. 2022). Spectral features have also distinguished comfortable from uncomfortably hot environments at the individual level (Wu et al. 2020). Together, these studies establish EEG spectral power as a sensitive marker of thermal state (Zhu et al. 2020; Liu et al. 2024, 2025). However, band power describes how strongly activity is expressed within predefined frequency ranges; even when evaluated across electrodes, it does not directly characterize the rapid succession of whole-scalp configurations, phase-based coordination between distributed signals, or the cortical generators of the observed changes. Consequently, spectral findings alone cannot determine whether temperature-related power changes reflect altered global brain-state dynamics, network synchronization, anatomically specific cortical responses, or a combination of these processes (Han et al. 2018). Earlier SEP and ERP studies showed that passive heat stress alters stimulus-evoked somatosensory processing and task-related cognitive responses (Nakata et al. 2015, 2017, 2019; Shibasaki et al. 2016; Shibasaki and Nakata 2026). Less is known about how ongoing EEG activity is organized during sustained thermal exposure in the absence of a task.

EEG microstate analysis, functional-connectivity analysis, and source imaging address these complementary levels of organization. Microstates segment continuous multichannel EEG into recurring, quasi-stable scalp topographies and quantify their duration, occurrence, coverage, and transition structure, thereby providing a millisecond-scale description of large-scale brain-state dynamics (Michel and Koenig 2018; Kleinert et al. 2024; Michel et al. 2024). Simultaneous EEG-fMRI research has further shown that canonical microstate topographies covary with distinct resting-state networks, supporting their use as electrophysiological indices of rapidly changing network configurations (Britz et al. 2010). Functional-connectivity measures assess statistical coupling between distributed neural signals; in particular, the weighted phase-lag index reduces sensitivity to zero-lag coupling caused by volume conduction and is more robust to noise than conventional phase-locking measures (Vinck et al. 2011; Sun et al. 2013). EEG source imaging complements these sensor-level approaches by estimating the intracranial generators that give rise to scalp-recorded potentials, although the solution remains dependent on electrode coverage, the head model, and inverse-method assumptions (Michel and Brunet 2019). Applying these methods together can therefore distinguish whether thermal exposure changes the temporal persistence of global states, the coordination among distributed signals, and the regional cortical expression of oscillatory activity (Liu et al. 2015). Such an integrated analysis remains uncommon in environmental-temperature research, which has largely emphasized spectral features and thermal-state classification, and thus offers a stronger basis for examining the spatiotemporal neural response to sustained thermal load (Nunneley et al. 2002).

To address this knowledge gap, the present study recorded EEG signals from participants exposed to different environmental temperatures and investigated temperature-related changes in EEG microstate dynamics, functional connectivity, and cortical source activity. Specifically, this study aimed to determine whether environmental temperature alters the temporal characteristics and transition patterns of EEG microstates, reorganizes functional interactions among brain regions, and modulates the cortical sources associated with thermal perception and cognitive regulation. By integrating these complementary measures, we sought to characterize temperature-associated differences in the spatiotemporal organization of ongoing EEG activity.

## Materials and Methods

### Subjects

An a priori power analysis was conducted using G*Power version 3.1.9.7, assuming an effect size of f = 0.5, α = 0.05, and power = 0.80. Fifteen healthy male volunteers aged 20–28 years participated in the study. Their height and body mass were 175 ± 5 cm and 70 ± 5 kg, respectively. Participants were eligible if they had no self-reported history of neurological or psychiatric disorders, alcohol abuse, or other medical conditions that could affect thermoregulation or EEG activity. They were instructed to maintain a regular sleep schedule for one week before the experiment and to refrain from consuming caffeine-containing products before each experimental session.

### Experimental Equipment

**Fig. 1:**
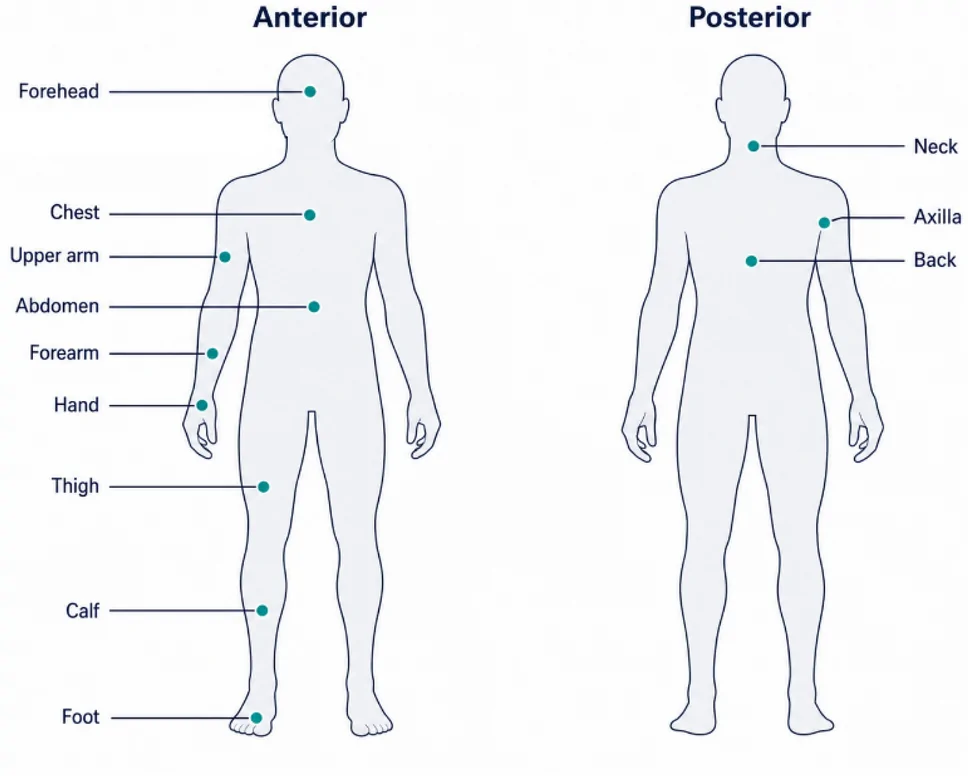
Placement of the temperature sensors during the experiment

To control for the influence of clothing insulation, all participants wore identical winter protective clothing during the experiment. The total thermal insulation of the clothing ensemble was 3.2 clo. EEG signals were recorded using a 32-channel wired EEG acquisition system manufactured by Neuroscan. The electrodes were positioned according to the international 10–20 system, and the signals were continuously sampled at 1000 Hz. The average of the left and right mastoid electrodes (M1 and M2) was used as the reference. Electrode–scalp impedance was maintained below 5 kΩ throughout the experiment. Local skin temperatures were measured using wireless iButton temperature loggers with a measurement accuracy of ±0.2 °C. Temperature data were recorded at 1-min intervals. The anatomical locations of the skin-temperature sensors are shown in Fig. 1. Core body temperature was measured using an ingestible telemetry sensor with a measurement accuracy of ±0.2 °C.

### Experimental Procedure

**Fig. 2:**
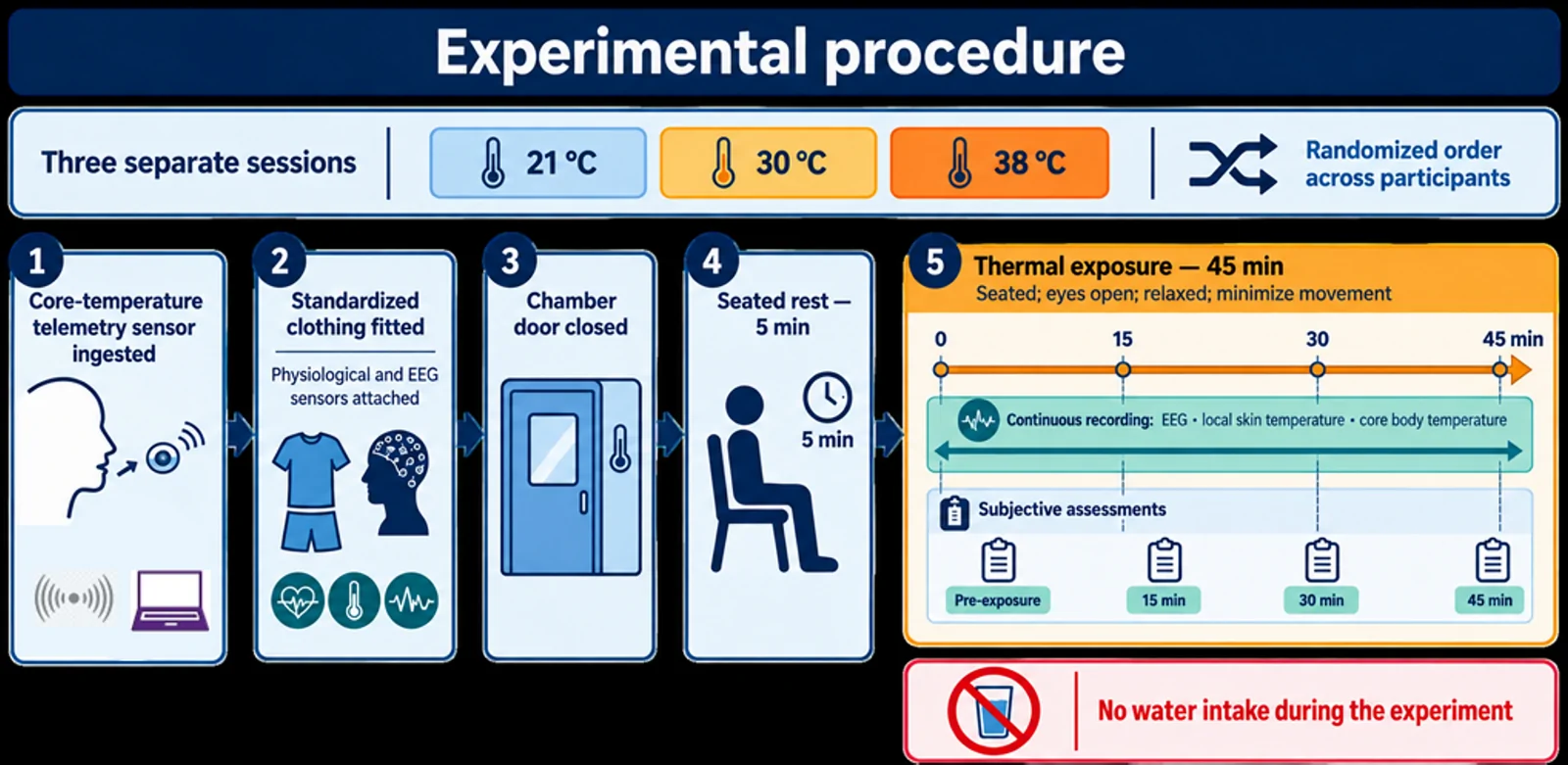
Experimental procedure

The experimental procedure is illustrated in Fig. 2. Each participant completed three separate experimental sessions at ambient temperatures of 21, 30, and 38 °C. Before each session, the participant ingested a core-temperature telemetry sensor. Then the experimenters fitted the standardized clothing and attached all physiological and EEG sensors. After the environmental-chamber door was closed, the participant remained seated at rest for an additional 5 min. Subsequently, the participant underwent a 45-min thermal exposure while remaining seated with his eyes open. He was instructed to remain relaxed and minimize unnecessary movements. EEG, local skin temperature, and core body temperature were continuously recorded throughout the exposure. Subjective assessments were conducted immediately before exposure and at 15, 30, and 45 min during exposure. Participants were not allowed to drink water during the experiment. The entire experiment was monitored in real time. To minimize potential order and carryover effects, the order of the three temperature conditions was randomized across participants, and consecutive sessions were separated by at least 15 days.

### Data Processing

#### Temperatures and Questionnaire

Local skin temperatures and core body temperature were analyzed to characterize participants’ physiological responses under the three environmental conditions. Mean skin temperature was calculated as the weighted average of local skin temperatures, with the weighting coefficient for each measurement site determined according to its relative contribution to the total body surface area, as shown in Eq. (1):

1 $$\begin{aligned}\mathrm{C}\mathrm{M}\mathrm{S}\mathrm{T}= & 0.15{T}_{\mathrm{a}\mathrm{b}\mathrm{d}\mathrm{o}\mathrm{m}\mathrm{e}\mathrm{n}}+0.15{T}_{\mathrm{c}\mathrm{h}\mathrm{e}\mathrm{s}\mathrm{t}}+0.09{T}_{\mathrm{b}\mathrm{a}\mathrm{c}\mathrm{k}}+0.09{T}_{\mathrm{u}\mathrm{p}\mathrm{p}\mathrm{e}\mathrm{r}\hspace{0.17em}\mathrm{a}\mathrm{r}\mathrm{m}}\\& +0.06{T}_{\mathrm{f}\mathrm{o}\mathrm{r}\mathrm{e}\mathrm{a}\mathrm{r}\mathrm{m}}+0.05{T}_{\mathrm{h}\mathrm{a}\mathrm{n}\mathrm{d}}+0.20{T}_{\mathrm{t}\mathrm{h}\mathrm{i}\mathrm{g}\mathrm{h}}+0.14{T}_{\mathrm{c}\mathrm{a}\mathrm{l}\mathrm{f}}+0.07{T}_{\mathrm{f}\mathrm{o}\mathrm{o}\mathrm{t}} \end{aligned}$$

where T represents the local skin temperature of the corresponding body part, and the sum of the weights is 1. CMST represents the calculated mean skin temperature (Houdas and Ring 1982).

#### EEG Processing

EEG preprocessing was performed in MATLAB R2021 using the EEGLAB toolbox (RRID:SCR_007292). Electrode coordinates were assigned according to the international 10–20 system. The 45-min continuous EEG signals were downsampled from 1000 to 200 Hz and band-pass filtered between 1 and 80 Hz, and a 50-Hz notch filter was applied to suppress power-line interference. The signals were subsequently re-referenced to the average across all EEG electrodes. Noisy or malfunctioning channels were identified and interpolated. Independent component analysis (ICA) was then performed to identify and remove components associated with ocular, muscular, cardiac, and other non-neural artifacts. The ICA components and the preprocessed EEG signals were visually inspected by an experienced researcher, and residual artifacts were manually rejected. The final 15 min of each 45-min exposure were selected for EEG analysis because the time courses of mean skin temperature and core temperature showed smaller changes during this interval than during the earlier exposure period. This window was chosen to assess EEG activity after the temperature-related physiological responses had become more established, while keeping the analyzed duration consistent across conditions. Surveillance video recordings from each 45-min exposure session were synchronized with the EEG timestamps and reviewed in 30-s epochs by two experienced researchers. Epochs showing possible behavioral signs of drowsiness, including yawning, visibly slowed blinking, or apparent involuntary changes in head posture, were flagged for further review. The corresponding EEG segments were examined for the presence of sleep spindles or K-complexes as corroborating evidence. An epoch was excluded only if it showed behavioral signs of drowsiness together with sleep spindles or K-complexes, and both researchers agreed on its exclusion. Identified epochs within the final 15-min analysis window were excluded before subsequent EEG analyses.

Sensor-level power spectral density (PSD) was estimated separately for each EEG channel using Welch’s method. PSD estimates were calculated from the preprocessed EEG sampled at 200 Hz using 800-sample (4-s) Hann windows with 400-sample overlap (50%), constant detrending, and a frequency resolution of 0.25 Hz. Absolute band power was obtained by trapezoidal integration of the PSD within the delta (1–4 Hz), theta (4–8 Hz), alpha (8–13 Hz), beta (13–30 Hz), and gamma (30–70 Hz) bands. Relative band power was calculated by dividing the absolute power within each frequency band by the total spectral power between 1 and 70 Hz. Thus, one relative-power value was obtained for each electrode and frequency band under each participant and temperature condition. Temperature effects on electrode-level relative power were assessed separately for each frequency-band-by-electrode combination using Friedman tests across the 21, 30, and 38 °C conditions.

EEG microstate analysis was performed in MATLAB using the MICROSTATELAB toolbox (Nagabhushan Kalburgi et al. 2024). EEG microstate analysis consisted of four main steps: GFP calculation, candidate-map selection, topographic clustering, and estimation of microstate temporal parameters. GFP was calculated at each time point as a measure of the global strength of the scalp electric field. Candidate scalp topographies were selected at local GFP maxima, with up to 1,200 peaks retained from each valid recording. The selected maps were normalized and pooled across participants and temperature conditions, and four polarity-invariant microstate templates were extracted using modified k-means clustering based on absolute spatial correlation. To assess the appropriate number of microstate classes, modified k-means clustering was repeated for k = 2–8. Model quality was evaluated using global explained variance (GEV) and the cross-validation (CV) criterion, for which lower values indicate a better balance between residual variance and model complexity. Because GEV necessarily increases as additional classes are introduced, its curve was inspected for diminishing incremental improvement rather than selecting the solution with the largest absolute GEV. The data-driven maps from the four-class solution were subsequently compared with canonical microstate maps A–D using polarity-invariant spatial correlation, quantified as the absolute Pearson correlation between corresponding electrode values. We repeated the polarity-invariant spatial matching using the Koenig templates, applying the same one-to-one assignment procedure as for the primary MetaMaps reference templates (Koenig et al. 2002, 2024). A one-to-one assignment between data-driven and canonical maps was obtained by maximizing the total absolute spatial correlation.

The common templates were back-fitted to each participant’s preprocessed EEG by assigning each time point to the template with the highest absolute spatial correlation. Temporal continuity was subsequently imposed using Besag smoothing with a factor of 10 and a 30-ms half-window. Segments shorter than 30 ms were reassigned to an adjacent state according to the spatial fitting error. For each microstate class, mean duration, occurrence rate, and time coverage were calculated. Transition probabilities were calculated from successive changes between different microstate classes and normalized according to the originating state. Coverage, occurrence, and mean duration were selected to characterize the proportion of time occupied by each state, its frequency of occurrence, and its temporal persistence, respectively.

Sensor-level functional connectivity was quantified using the weighted phase-lag index (wPLI) to examine whether ambient-temperature conditions were associated with differences in phase-lagged synchronization between EEG signals. We prioritized this measure because our connectivity analysis focused on phase relationships, complementing the separate analysis of relative spectral power. Its reduced sensitivity to zero-lag coupling potentially arising from volume conduction further motivated its use for sensor-level analysis (Vinck et al. 2011). Preprocessed EEG data were divided into 4-s epochs and filtered into the delta, theta, alpha, beta, and gamma bands. The analytic signal of each channel was obtained using the Hilbert transform, and wPLI was calculated for every unique pair of EEG channels based on the imaginary component of their cross-spectral relationship. The resulting values were averaged across epochs to generate a symmetric channel-by-channel connectivity matrix for each participant under each temperature condition, with diagonal elements set to zero. Global connectivity strength was defined as the mean wPLI across all unique channel pairs. Temperature effects on global wPLI were examined using Friedman tests, with false discovery rate correction applied across frequency bands. Significant omnibus effects were followed by pairwise Wilcoxon signed-rank tests with Holm correction. For regional connectivity analysis, electrodes were assigned to five regions: frontal(Fp1, Fp2, F3, F4, F7, F8, Fz), central(FC1, FC2, FC5, FC6, C3, C4, Cz), temporal(FT9, FT10, T7, T8, TP9, TP10), parietal(CP1, CP2, CP5, CP6, P3, P4, P7, P8, Pz), and occipital(O1, O2, Oz). For each participant, temperature condition, and frequency band, within-region connectivity was calculated as the mean wPLI across all unique pairs of distinct electrodes within that region. Between-region connectivity was calculated as the mean wPLI across all electrode pairs spanning the two regions. This yielded 15 regional connectivity values per frequency band: five within-region and ten between-region values. These values were displayed as symmetric regional connectivity heatmaps using a common color scale; no connectivity threshold was applied for visualization.

EEG source localization was performed in MNE-Python (RRID:SCR_005972) using the FreeSurfer fsaverage template (RRID:SCR_001847). Standard 10–20 electrode coordinates were used because individual electrode coordinates and MRI scans were unavailable. A single EEG-to-fsaverage transformation was estimated using anatomical fiducials followed by iterative closest-point fitting and applied to all participants. An ico-4 cortical source space comprised 5,124 vertices (2,562 per hemisphere). The EEG forward solution was computed using the fsaverage three-layer boundary element model (BEM); all 5,124 vertices were retained.

An ad hoc diagonal noise covariance matrix was used to construct a depth-weighted minimum-norm inverse operator (depth weighting = 0.8). Source orientations were fixed normal to the cortical surface, yielding one scalar source per vertex. Dynamic statistical parametric mapping (dSPM) was applied with a regularization parameter of λ^2^ = 1/9, corresponding to an assumed signal-to-noise ratio of 3.

Source power was calculated from the artifact-screened EEG data sampled at 200 Hz. Cortical signals were summarized within the 68 Desikan–Killiany regions of interest (ROIs) using sign-flip–corrected averaging (mean_flip) to reduce cancellation across source orientations. Power spectral density was estimated using Welch’s method with 4-s Hann windows and 50% overlap, giving a frequency resolution of 0.25 Hz. Band power was obtained by integrating the spectrum over delta, theta, alpha, beta, and gamma. Relative source power at each ROI or cortical vertex was defined as power in the specified band divided by total power from 1 to 70 Hz at the same location.

#### Statistical Analysis

Skin temperature, core body temperature, and questionnaire outcomes were summarized descriptively for each temperature condition and were not subjected to inferential statistical testing. EEG statistical analyses were performed in Python using SciPy and custom analysis scripts. Differences among the 21, 30, and 38 °C conditions were evaluated using Friedman tests.

For sensor-level relative spectral power, separate Friedman tests were conducted for each frequency-band–electrode combination, and the Benjamini–Hochberg false discovery rate (FDR) correction was applied across 160 comparisons comprising five frequency bands and 32 electrodes. Source-level relative power was similarly evaluated for each frequency-band–ROI combination, with FDR correction across 340 comparisons comprising five frequency bands and 68 cortical ROIs. Global mean weighted phase-lag index (wPLI) was compared across temperatures in each frequency band, with FDR correction across the five bands. For regional wPLI, separate Friedman tests were conducted across the three temperature conditions for each combination of frequency band and regional connection. Benjamini–Hochberg FDR correction was applied jointly across all 75 tests, comprising five frequency bands and 15 electrode-defined regional connections (five within-region and ten between-region connections). Microstate coverage, mean duration, and occurrence were analyzed separately, with FDR correction applied across the four microstate classes within each parameter.

When an omnibus temperature effect was significant, pairwise comparisons between temperature conditions were performed using Wilcoxon signed-rank tests, with Holm correction across the three contrasts. Kendall’s W was reported as the effect size for Friedman tests, and paired rank-biserial correlation (r_rb_) was reported for Wilcoxon comparisons. Microstate transition probabilities were summarized descriptively and were not interpreted as edge-wise inferential findings. All tests were two-sided, with FDR-adjusted (q<0.05) or Holm-adjusted (p<0.05) considered statistically significant.

## Results

### Skin Temperature and core Temperature

**Fig. 3:**
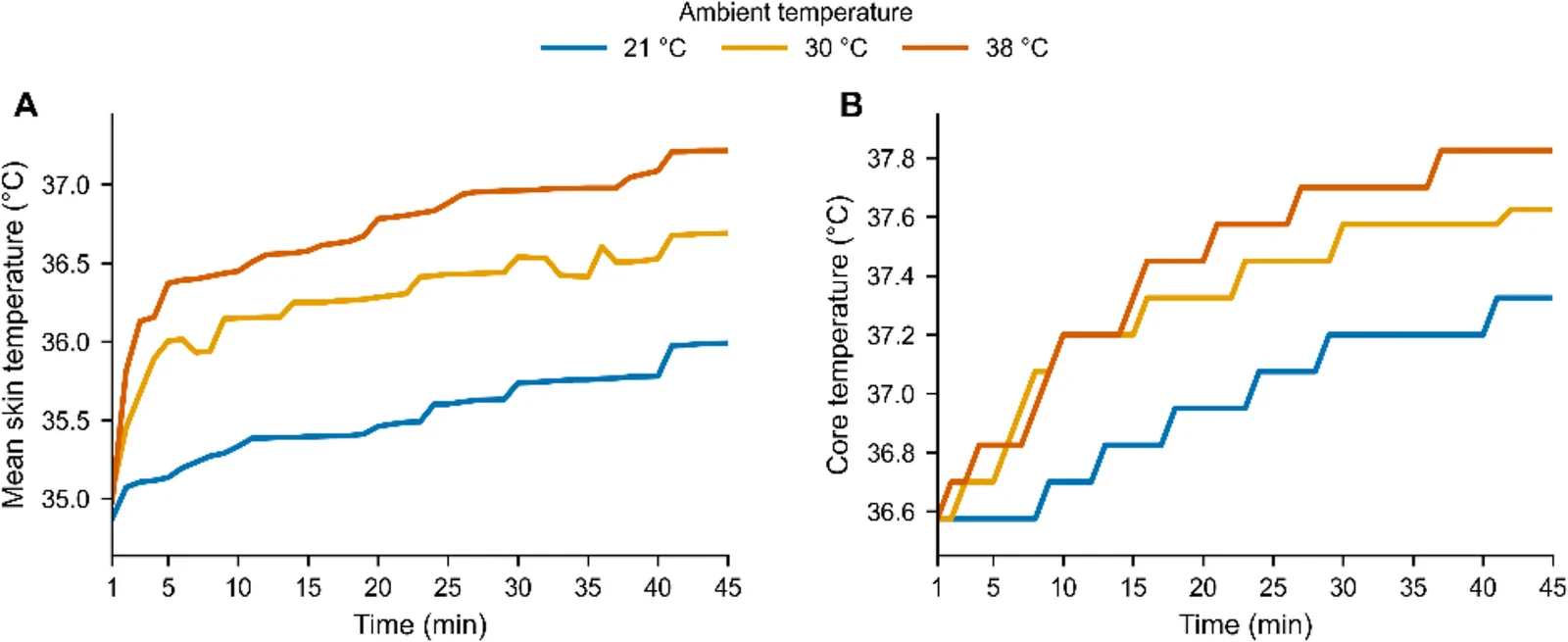
Temporal changes in physiological temperature responses under the three ambient-temperature conditions. (**A**) Mean skin temperature and (**B**) core body temperature during the 45-min exposure at 21, 30, and 38 °C

Figure 3 illustrates the temporal changes in mean skin temperature and core body temperature under the three ambient-temperature conditions. As shown in Fig. 3A, the initial mean skin temperatures were comparable across the three conditions, with an overall value of 34.97 ± 0.09 °C. Mean skin temperature increased over time under all conditions, although the magnitude and temporal pattern of the increase differed. At 21 °C, mean skin temperature increased gradually and reached a plateau of approximately 35.78 ± 0.01 °C after 30 min. At 30 °C, it continued to increase until approximately 37 min and subsequently approached a plateau of 36.52 ± 0.01 °C. At 38 °C, mean skin temperature increased throughout the exposure period, reaching 37.09 ± 0.02 °C at 40 min. Figure 3B shows the temporal changes in core body temperature. The initial core body temperatures were comparable across the three conditions, with an overall value of 36.58 ± 0.03 °C. At 21 °C, core body temperature increased gradually and reached 37.33 ± 0.01 °C by the end of the exposure. Under the 30 and 38 °C conditions, similar increasing trends were observed during the first 14 min. Thereafter, the increase was less pronounced at 30 °C, with core body temperature reaching 37.63 ± 0.01 °C at the end of the exposure. In contrast, core body temperature continued to increase more rapidly at 38 °C and reached 37.83 ± 0.02 °C.

### Questionnaire

**Fig. 4:**
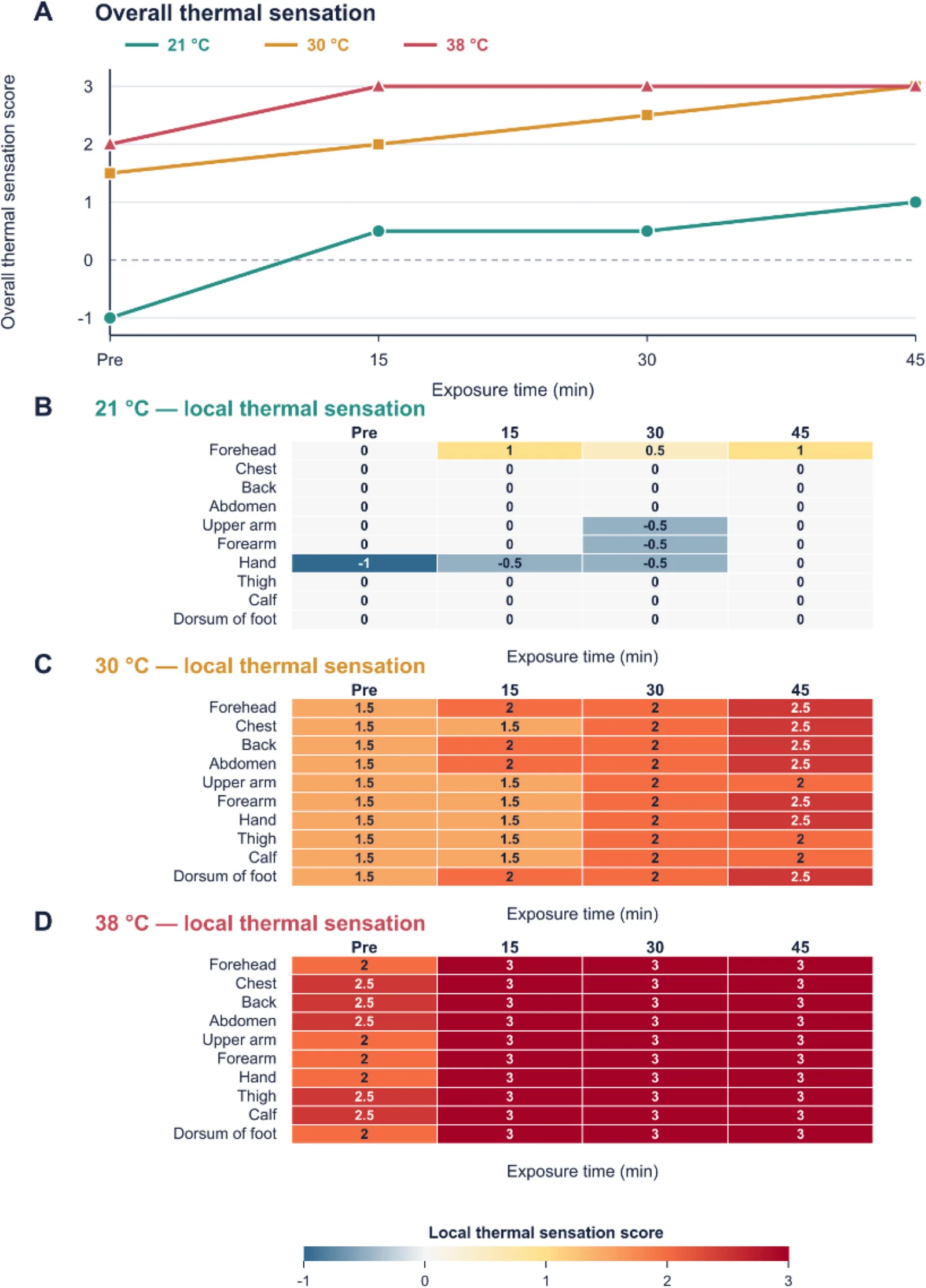
Whole-body and local thermal-sensation responses under the three ambient-temperature conditions. (**A**) Changes in overall thermal sensation scores during exposure. (**B–D**) Local thermal sensation scores at different body sites under the 21, 30, and 38 °C conditions, respectively. Values within the heatmaps indicate the corresponding thermal sensation scores

Figure 4 shows the whole-body and local thermal-sensation votes under the three ambient-temperature conditions. At 21 °C, the whole-body thermal sensation was generally rated between neutral and slightly warm, with mean votes ranging from approximately 0.5 to 1.0. However, exposed body regions showed relatively cooler sensations; in particular, the hands, which were not covered by clothing, were rated as slightly cool, with a mean vote of approximately −0.5. At 30 °C, whole-body thermal-sensation votes increased over the course of exposure from approximately 1.5 to 3.0. At 38 °C, all assessed body regions were perceived as warm to hot, with thermal-sensation votes ranging from approximately 2.0–2.5 to 3.0. Thermal-sensation votes generally shifted toward the warmer end of the scale with increasing exposure time under all three conditions. Overall, subjective thermal sensation increased with both ambient temperature and exposure duration, and the highest whole-body and local thermal-sensation votes occurred at 38 °C.

### EEG Power

Across the full 45-min recordings, the mean numbers of 30-s epochs identified as showing overt signs of drowsiness were 1.20, 1.33, and 1.47 per participant at 21, 30, and 38 °C, respectively. These counts did not differ significantly across temperature conditions (Friedman test, p = 0.724). Within the final 15-min analysis window, the corresponding mean counts were 0.47, 0.53, and 0.60 epochs per participant. After these epochs were excluded, the omnibus temperature effects on relative delta, beta, and gamma power remained significant after FDR correction (delta and beta: q < 0.001; gamma: q = 0.038), and the direction of the differences across temperature conditions was preserved.

**Fig. 5:**
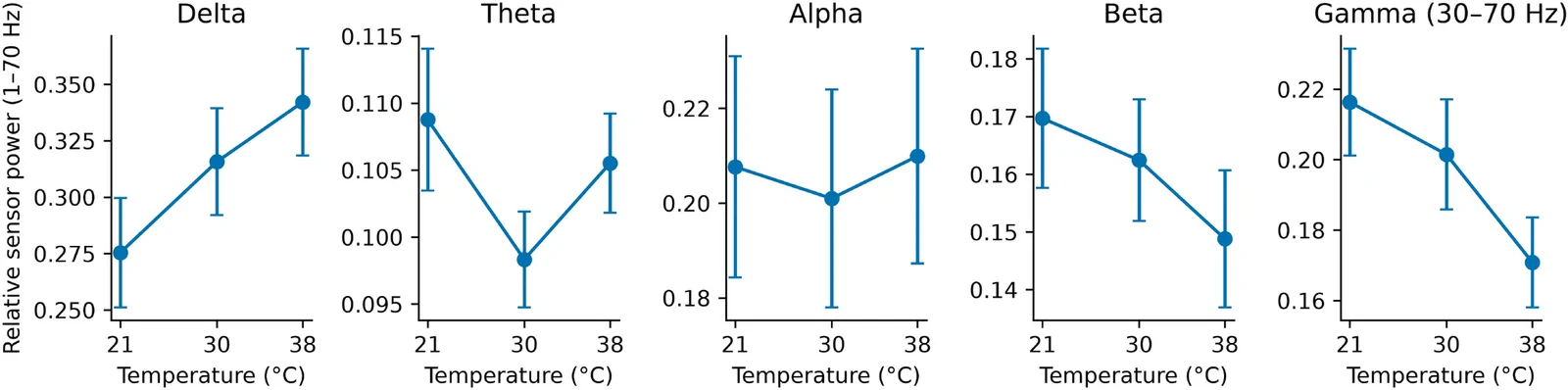
Relative EEG spectral power under the three ambient-temperature conditions. Relative power was normalized to total power from 1 to 70 Hz and calculated for the delta, theta, alpha, beta, and gamma bands at 21, 30, and 38 °C

Relative EEG spectral power, normalized to total power from 1 to 70 Hz, is shown in Fig. 5. Global delta power increased across the 21, 30, and 38 °C conditions (0.275, 0.316, and 0.342, respectively; χ^2^=19.15, FDR-adjusted q<0.001, Kendall’s W=0.638). Delta power was higher at 30 °C than at 21 °C (Holm-adjusted p=0.0026, r_rb_=0.86) and at 38 °C than at 21 °C (Holm-adjusted p<0.001, r_rb_=0.93); the difference between 30 and 38 °C was not significant. Beta power also differed among conditions (χ^2^=18.55, q<0.001, W=0.618) and was lower at 38 °C than at both 21 and 30 °C (both Holm-adjusted p<0.001). For the gamma band, relative power decreased from 0.216 at 21 °C to 0.201 at 30 °C and 0.171 at 38 °C (χ^2^=7.56, q=0.0380, W=0.25). Gamma power was lower at 38 °C than at 21 °C (Holm-adjusted p=0.001, r_rb_=−0.88), whereas the other pairwise comparisons were not significant after Holm correction. No overall theta- or alpha-band effect remained significant after FDR correction

**Fig. 6:**
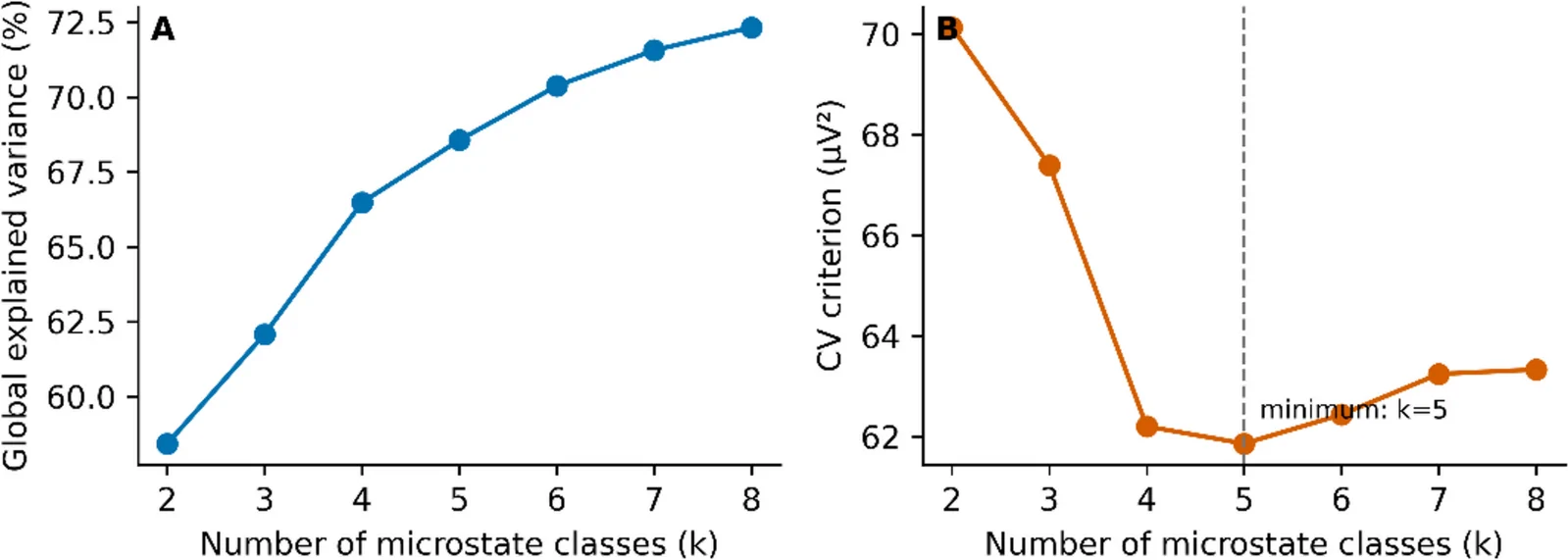
Model selection of the EEG microstate solutions. (A) Global explained variance (GEV) for clustering solutions containing 2–8 microstate classes. (B) Cross-validation criterion across the same candidate solutions

### Microstates

Model-selection results are shown in Fig. 6. GEV increased from 58.4% at k = 2 to 66.5% at k = 4 and continued to increase more gradually thereafter, reaching 72.3% at k = 8. The largest improvements occurred before or at k = 4, followed by diminishing gains for additional classes. CV decreased markedly between k = 2 and k = 4 and reached its lowest value at k = 5 (approximately 61.9 μV2), only slightly below the value obtained for k = 4 (approximately 62.2 μV2). Taken together, the pronounced GEV improvement up to four classes, the small CV difference between k = 4 and k = 5, and the interpretability of the canonical A–D configuration supported retaining the parsimonious four-class solution. 

**Fig. 7:**
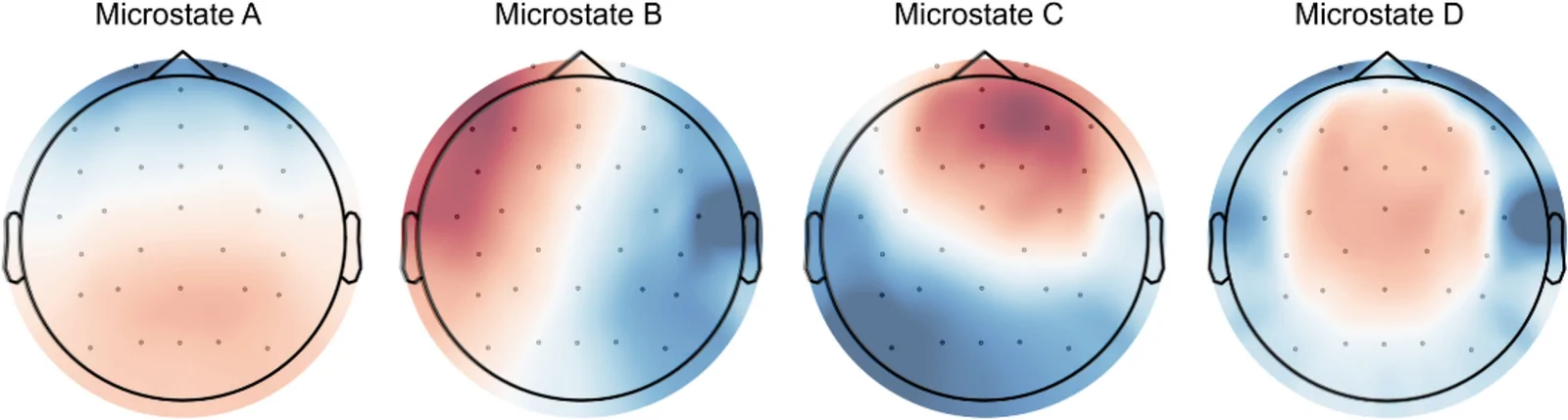
Group-level EEG microstate topographies pooled across the three conditions

Four polarity-invariant microstate templates were jointly extracted from the global field power peaks of all artifact-free EEG recordings and were labeled as microstates A–D (Fig. 7). The four classes exhibited distinct and relatively stable scalp-potential topographies and were used as common spatial templates for the subsequent calculation of microstate temporal parameters and transition characteristics.

Polarity-invariant matching to the MetaMaps reference templates yielded absolute spatial correlations of 0.726, 0.794, 0.793, and 0.692 for microstates A–D, respectively. Additional matching to the Koenig (2002) templates yielded corresponding correlations of 0.747, 0.703, 0.754, and 0.653, with the same one-to-one class assignment across the two reference sets. The four-class solution explained 64 ± 8%, 65 ± 7%, and 72 ± 8% of the global variance under the 21, 30, and 38 °C conditions, respectively. Although the mean GEV was numerically higher at 38 °C, no statistically significant difference was observed among the three temperature conditions (p>0.05).

**Fig. 8:**
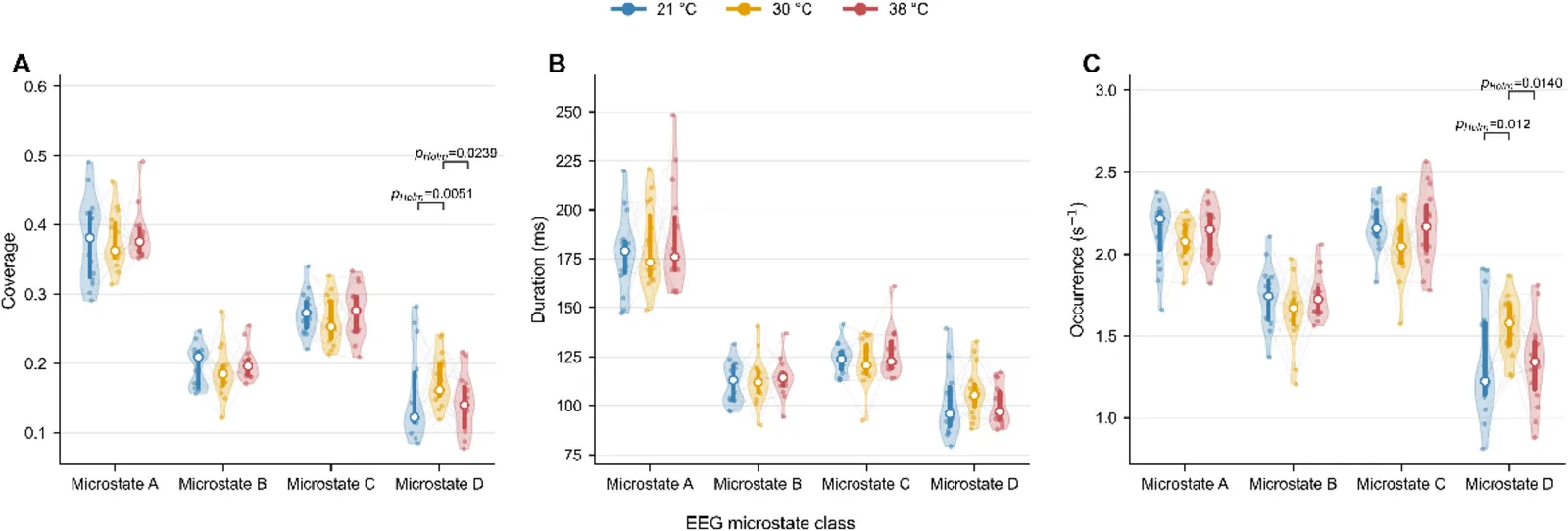
Temporal parameters of EEG microstates under the three ambient-temperature conditions. (**A**) Coverage, (**B**) mean Duration, and (**C**) Occurrence rate

Microstate D coverage differed across temperatures, with values of 0.145, 0.178, and 0.150 at 21, 30, and 38 °C, respectively (χ^2^=12.88, FDR-adjusted q=0.0064, W=0.429). Coverage was higher at 30 °C than at 21 °C (${p}_{\mathrm{H}\mathrm{o}\mathrm{l}\mathrm{m}}$=0.0051, r_rb_=0.92) and 38 °C (${p}_{\mathrm{H}\mathrm{o}\mathrm{l}\mathrm{m}}$=0.0239, r_rb_=0.74). Microstate D occurrence also differed across temperatures (1.30, 1.57, and 1.37 s⁻^1^; χ^2^=12.69, q=0.0070, W=0.423), with a higher rate at 30 °C than at 21 °C (${p}_{\mathrm{H}\mathrm{o}\mathrm{l}\mathrm{m}}$=0.012, r_rb_=0.76) and 38 °C (${p}_{\mathrm{H}\mathrm{o}\mathrm{l}\mathrm{m}}$=0.014, r_rb_=0.70). No duration effect survived FDR correction (all q≥0.105) (Fig. 8).

**Fig. 9:**
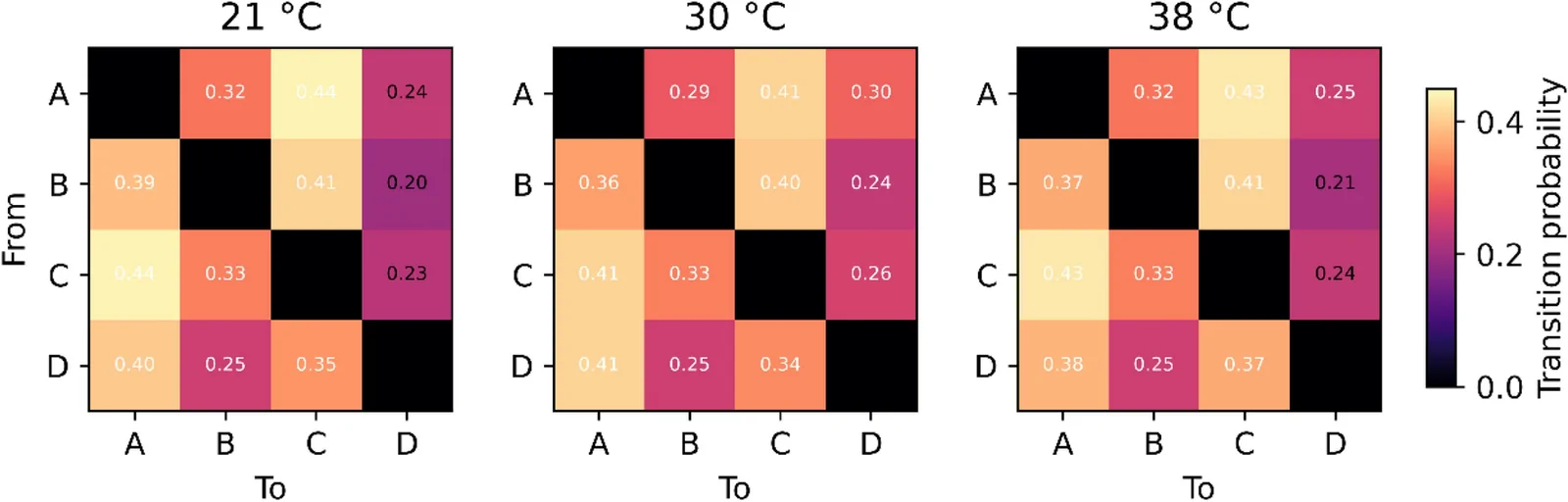
Transition probabilities among EEG microstates **A–D** under the three ambient-temperature conditions

Microstate transition probability matrices are presented in Fig. 9. Transition patterns were broadly similar across temperatures, with only modest descriptive differences. Compared with 21 °C, the mean transition probabilities of A→D, B→D, and C→D were slightly higher at 30 °C, whereas those of A→C, B→A, and C→A were slightly lower, suggesting an increased tendency for the microstate sequence to transition toward microstate D at 30 °C. Transition probabilities at 38 °C were generally comparable to those at 21 °C, except for a slight increase in D→C and a slight decrease in D→A.

### Functional Connectivity

Global mean delta-band wPLI increased across the 21, 30, and 38 °C conditions (0.151 ± 0.064, 0.181 ± 0.062, and 0.194 ± 0.094, respectively). A Friedman test indicated a significant overall difference among temperature conditions (χ^2^=12.64, FDR-adjusted q=0.0090, W=0.421). Delta-band wPLI was higher at 38 °C than at 21 °C (Holm-adjusted p=0.0139, r_rb_=0.79). No overall temperature effect was significant for theta, alpha, beta, or gamma connectivity after FDR correction.

**Fig. 10:**
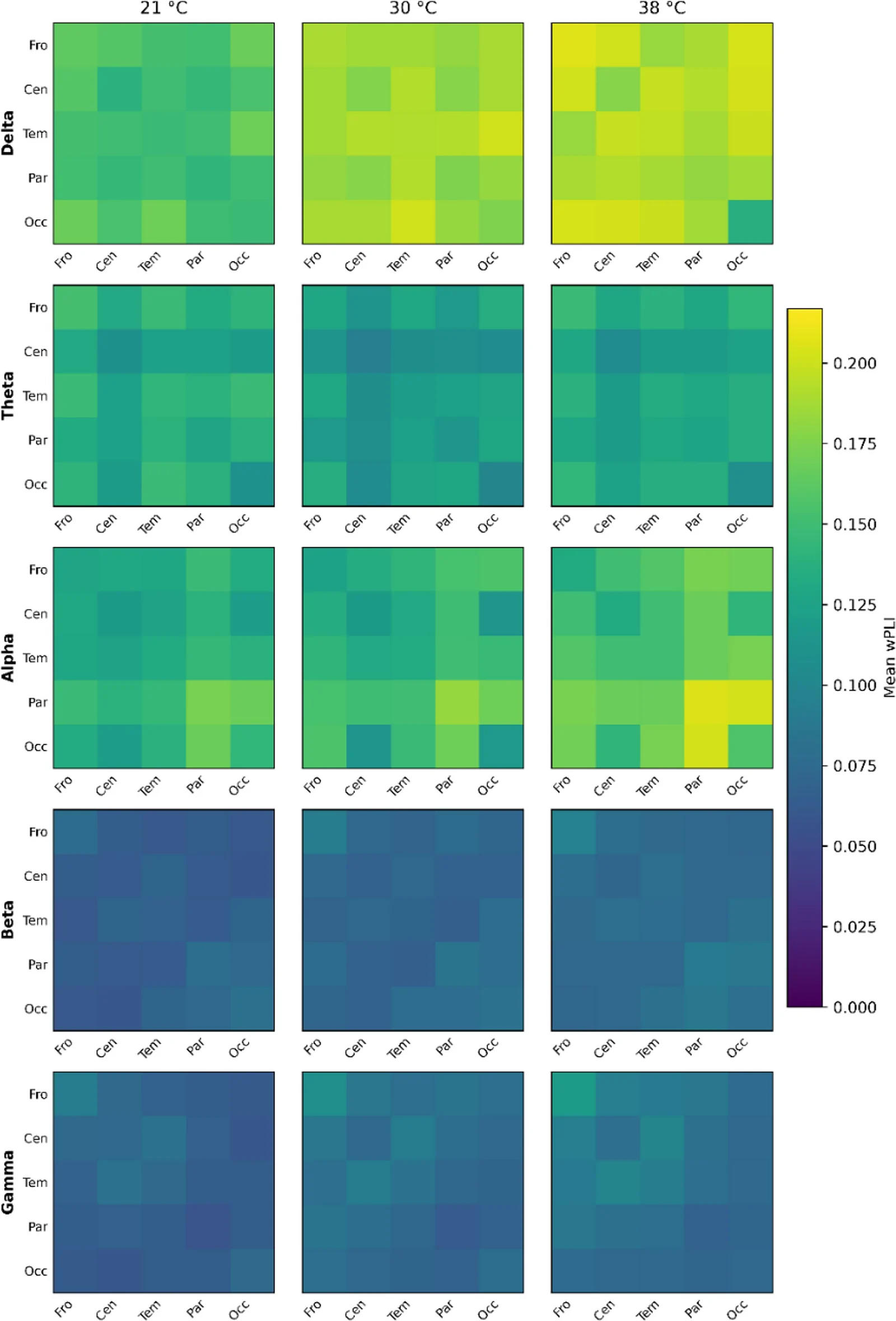
Sensor-level regional functional connectivity under the three ambient-temperature conditions. Heatmaps show the mean weighted phase lag index (wPLI) for each pair of electrode-defined regions across the five frequency bands at 21, 30, and 38 °C. The shared color bar represents mean wPLI magnitude on the same scale for all panels; colors do not indicate relative link strength or statistical significance. Diagonal cells represent the mean connectivity between distinct electrodes within the same region, whereas off-diagonal cells represent the mean connectivity between electrodes in different regions. Abbreviations: Fro, frontal; Cen, central; Tem, temporal; Par, parietal; Occ, occipital

Regional sensor-level connectivity showed temperature-related differences predominantly in the delta band (Fig. 10). Of the 75 omnibus tests spanning five frequency bands and 15 electrode-defined regional connections, nine remained significant after FDR correction across all 75 tests: seven in the delta band, one in the beta band, and one in the gamma band (Table 1). Significant delta-band effects involved within-central, within-temporal, and within-parietal connectivity, together with central–temporal, central–parietal, frontal–temporal, and temporal–parietal connectivity (q = 0.0043–0.0483). Across these seven connections, mean wPLI values ranged from 0.138–0.153 at 21 °C, 0.175–0.192 at 30 °C, and 0.178–0.198 at 38 °C. All seven showed numerically higher mean connectivity under both warmer conditions than at 21 °C. Significant omnibus effects were also observed for within-temporal beta connectivity (mean wPLI: 0.067, 0.070, and 0.078 at 21, 30, and 38 °C, respectively; q = 0.0483) and temporal–parietal gamma connectivity (0.064, 0.071, and 0.079, respectively; q = 0.0314). No regional connection in the theta or alpha band survived FDR correction.

**Table 1:** Regional sensor-level connectivity showing significant omnibus temperature effects after FDR correction

Frequency band	Region 1	Region 2	χ^2^	Kendall’s W	q
delta	Central	Central	18.151	0.605	0.004
delta	Temporal	Temporal	19.170	0.639	0.004
delta	Central	Temporal	16.906	0.564	0.005
delta	Temporal	Parietal	12.340	0.411	0.031
gamma	Temporal	Parietal	12.491	0.416	0.031
beta	Temporal	Temporal	10.302	0.343	0.048
delta	Central	Parietal	10.302	0.343	0.048
delta	Frontal	Temporal	11.019	0.367	0.048
delta	Parietal	Parietal	10.302	0.343	0.048

### Global and Regional Relative Source Power

**Fig. 11:**
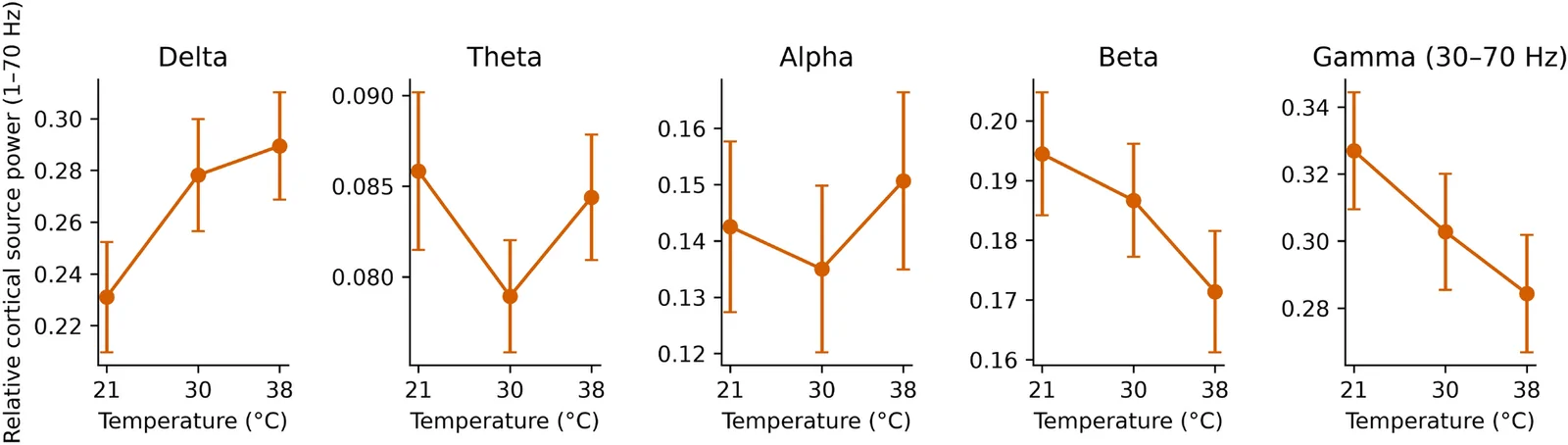
Whole-cortex mean relative source power under the three ambient-temperature conditions

Figure 11 shows the whole-cortex mean relative source power across frequency bands under the three ambient-temperature conditions. Source-level results were broadly consistent with the sensor-level spectral findings. Whole-cortex delta source power increased across temperatures (0.231, 0.278, and 0.289 at 21, 30, and 38 °C, respectively; χ^2^=15.88, FDR-adjusted q=0.0015, W=0.529). Delta source power was higher at 30 °C than at 21 °C (Holm-adjusted p=0.0011, r_rb_=0.89) and at 38 °C than at 21 °C (Holm-adjusted p<0.001, r_rb_=0.92). Beta source power decreased at 38 °C (χ^2^=14.88, q=0.0015, W=0.50), with significant differences between 21 and 38 °C (Holm-adjusted p<0.001, r_rb_=−0.843) and between 30 and 38 °C (Holm-adjusted p=0.0078, r_rb_ =−0.700). Mean relative gamma source power band decreased from 0.327 at 21 °C to 0.303 at 30 °C and 0.284 at 38 °C, but its overall temperature effect did not remain significant after FDR correction. A total of 111 frequency-band-by-region comparisons remained significant after FDR correction.

**Fig. 12:**
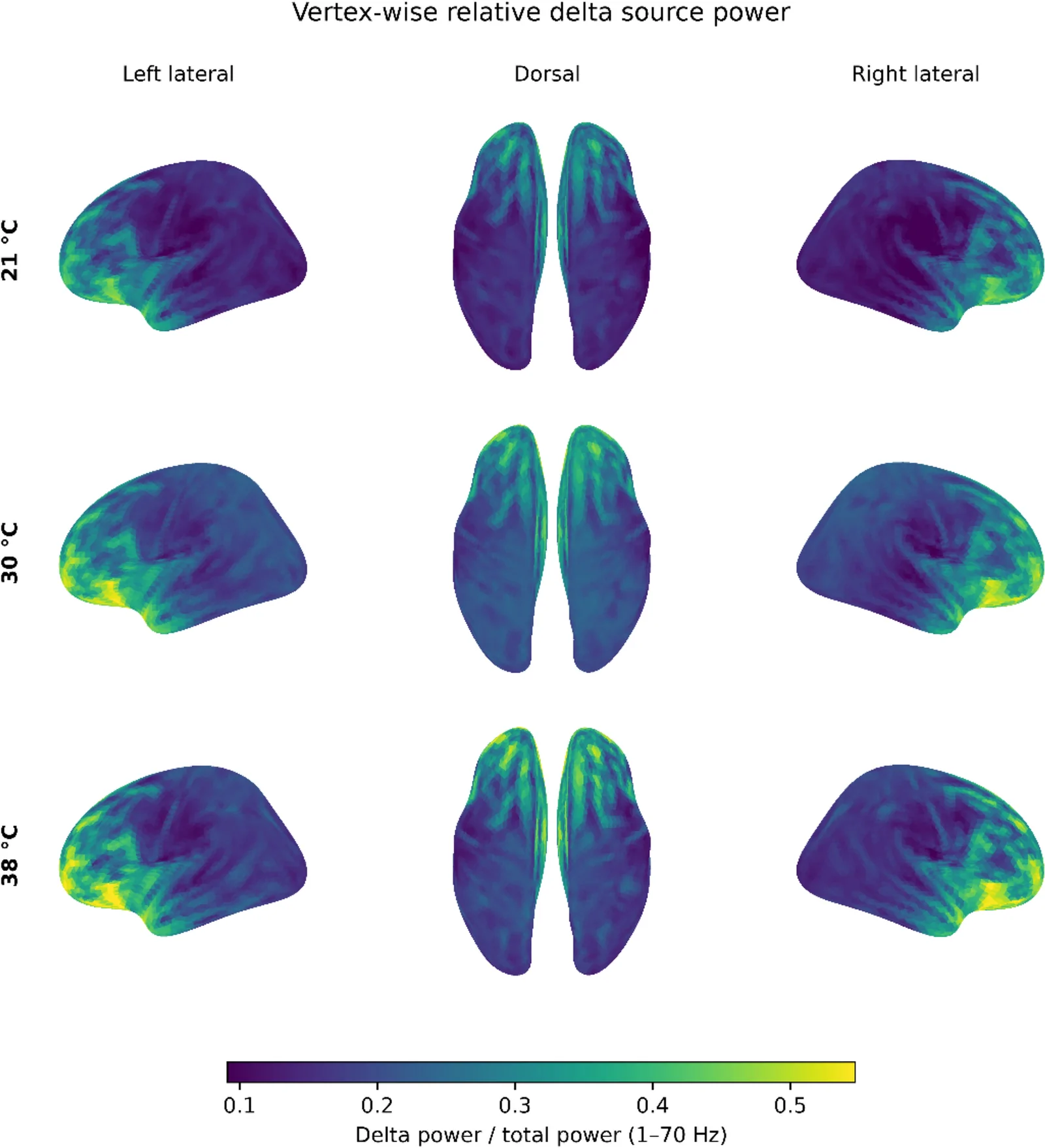
Vertex-wise cortical distribution of relative delta-band (1–4 Hz) source power across 21, 30, and 38 °C. EEG source activity was estimated using a fixed-orientation, depth-weighted dSPM inverse solution on the fsaverage ico-4 cortical mesh with a three-layer BEM forward model. Vertex-wise maps for the theta, alpha, beta, and gamma bands under all three temperature conditions are provided in Appendix 1 Fig. A1

Source-space analysis using the revised BEM model revealed temperature-related differences in relative source power (Fig. 12). Delta showed the most widespread regional effects, with 48 of 68 cortical ROIs significant after FDR correction across all band-by-ROI tests. Significant regional effects were also found in 9 theta, 12 alpha, 31 beta, and 11 gamma ROIs.

## Discussion

The principal finding was that ambient-temperature conditions were associated with differences in EEG spectral power, microstate temporal parameters, phase-lagged connectivity, and estimated cortical source power. The clearest signature of the 38 °C condition was increased relative delta power, reduced beta and gamma power, and stronger delta-band wPLI relative to 21 °C. In contrast, microstate D coverage and occurrence showed a non-linear response, peaking at 30 °C, whereas no microstate duration effect survived FDR correction. These neural changes occurred alongside progressive increases in mean skin temperature, core body temperature, and thermal-sensation votes. Together, the findings describe a shift toward lower-frequency relative power and increased delta-band wPLI, alongside a distinct, non-monotonic change in microstate D expression. The non-monotonic microstate D response at 30 °C further indicates that the neural response cannot be reduced to a linear function of ambient temperature(Omidvar and Kim 2022).

The spectral findings are broadly consistent with previous evidence of EEG slowing during heat strain. Nybo and Nielsen reported a progressive shift toward lower EEG frequencies during exercise-induced hyperthermia, and Lang et al. found that, across indoor exposures from 26 to 37 °C, greater delta activity was associated with warmer sensation and poorer thermal comfort and neurobehavioral performance (Nybo and Nielsen 2001; Lang et al. 2022). Our observation of increased relative delta power together with reduced beta and gamma power at 38 °C extends this pattern to a passive, seated exposure with high clothing insulation. Importantly, the present data do not establish cognitive impairment because cognitive outcomes were not measured. The lower-frequency shift may be compatible with changes in arousal, but the absence of independent vigilance and fatigue measures prevents a firm functional interpretation. Differences from studies using brief local thermal stimulation are also informative (Chang et al. 2005).

The microstate findings add a temporal dimension that cannot be captured by conventional spectral analysis. Following temporal smoothing, microstate D coverage and occurrence were higher at 30 °C than at both 21 and 38 °C, whereas no microstate class showed a significant temperature-related difference in mean duration after FDR correction. Because microstate coverage is jointly determined by occurrence and duration, the concurrent increase in coverage and occurrence, in the absence of a duration effect, suggests that the greater expression of microstate D at 30 °C was primarily driven by more frequent entries into this state rather than by prolonged individual episodes. Consistent with this interpretation, the transition matrices showed small descriptive increases in transitions toward microstate D at 30 °C, although individual transition pathways were not subjected to inferential testing. The return of microstate D coverage and occurrence at 38 °C to levels comparable with those at 21 °C suggests a non-linear, condition-specific response rather than two successive stages of thermal adaptation. In contrast, several delta-band regional wPLI connections were elevated relative to 21 °C at both 30 and 38 °C. Thus, the microstate D peak at 30 °C and the delta connectivity pattern do not support a discrete thermal disruption threshold or a switch to global low-frequency synchronization specifically at 38 °C. Because the present study used data-derived common templates and did not directly measure the cognitive processes potentially represented by microstate D, the most conservative conclusion is that the 30 °C condition selectively increased the temporal expression of this topographic state.

The connectivity results converge with this interpretation, but they also require careful qualification. Delta-band global wPLI was significantly higher at 38 °C than at 21 °C, indicating stronger phase-lagged coordination at the same low frequencies that gained relative power. Previous thermal-stimulation studies have likewise shown that temperature changes can alter distributed EEG coupling, although the affected bands and directions depend on the paradigm. Chang et al. reported increased coherence in delta and alpha bands during local thermal stimulation, while Santos Cuevas et al. found warm-cold differences in directed theta- and alpha-band interactions, particularly over frontal and temporal regions (Chang et al. 2005; Santos Cuevas et al. 2024). Increased wPLI indicates stronger phase-lagged coupling but does not, by itself, establish changes in network integration, differentiation, or compensatory function. Temperature-related changes in regional connectivity were concentrated in the delta band, involving central, temporal, and parietal within-region connections and several connections between regions. Delta-band wPLI was generally higher at 30 °C and 38 °C than at 21 °C. These findings suggest altered coordination of low-frequency activity under warmer conditions, alongside the observed increase in delta power. Connectivity was estimated between scalp electrodes grouped by region, so the reported regional differences should not be interpreted as direct connectivity between the corresponding cortical lobes. wPLI reduces sensitivity to zero-lag coupling, but sensor-level estimates may still be affected by signal mixing. Source-space connectivity analysis with appropriate control for source leakage would help assess the anatomical specificity and robustness of these findings (Siems et al. 2016; Rolle et al. 2022; Kida et al. 2023). Phase-based and amplitude-based connectivity capture complementary aspects of statistical coupling. Whereas wPLI characterizes phase-lagged synchronization, amplitude-envelope correlation assesses covariation in the amplitude envelopes of oscillatory signals (Siems and Siegel 2020). Our analysis of relative band power does not measure this interregional amplitude covariation. The present findings therefore characterize phase-based coupling and do not establish whether amplitude-based connectivity changes similarly across temperature conditions. Future studies could compare wPLI with leakage-corrected amplitude-envelope connectivity to assess whether the observed temperature-associated patterns extend across these complementary measures.

Source localization provides an anatomical context for this cross-scale pattern. Temperature-sensitive delta changes were widespread in the occipital and parietal cortices and the posterior cingulate, whereas beta reductions at 38 °C involved sensorimotor, parietal, and temporo-occipital regions. Although the overall temperature effect on mean source-level gamma power did not survive FDR correction, region-specific reductions were observed in several cortical ROIs. These regions have been associated with visual, sensorimotor, and internally oriented processing in previous research; however, the present source-power findings do not establish changes in these functions (Valt et al. 2024). The posterior cingulate/precuneus finding is particularly consistent with fMRI evidence that passive hyperthermia alters activity and connectivity in medial prefrontal, anterior cingulate, posterior cingulate/precuneus, and fusiform regions, and that head cooling partly normalizes these changes (Xue et al. 2018). More severe hyperthermia has also been associated with reduced sensorimotor cortical activation and impaired executive function (Tan et al. 2024).

Considered together, the findings indicate temperature-associated differences in several complementary aspects of ongoing EEG activity, without establishing their underlying cognitive or regulatory mechanisms. Human neuroimaging studies have implicated the somatosensory, insular, cingulate, parietal, and prefrontal cortices in thermal sensation, interoceptive processing, and thermoregulatory responses, although the direction and functional meaning of regional changes vary across paradigms (Farrell 2016; (“Bud”) Craig 2018). As skin and core temperatures and perceived warmth increased, cortical activity in the present study shifted toward lower frequencies, delta-band coordination strengthened, microstate expression changed, and posterior and sensorimotor source activity was redistributed. Similar EEG slowing has been reported under hot and humid conditions, but its relationship with cognitive performance is not always consistent (Zhu et al. 2020). The peak in microstate D at 30 °C may therefore indicate a non-linear reconfiguration under moderate thermal load rather than progressive deterioration. This interpretation is broadly consistent with evidence that cognitive responses to heat may be non-linear and task dependent (Schmit et al. 2017; Malcolm et al. 2018), but it does not establish that microstate D specifically represents thermal adaptation. The increased expression of microstate D at 30 °C identifies a change in the temporal occurrence of a spontaneous topography, while regional delta-band wPLI differences characterize phase-lagged coupling between electrode signals and dSPM maps depict estimated changes in the cortical distribution of delta power. These observations cannot be inferred from stimulus-locked SEP/ERP amplitudes and latencies alone. Conversely, the present design cannot determine whether sensory processing or task-related cognition was altered, as no corresponding stimuli or tasks were administered.

Several limitations temper the interpretation of these findings. The study included only 15 healthy young men. Although an a priori power calculation supported this sample size for a large effect, it does not ensure adequate power for smaller effects or support generalization across sexes and age groups. Sex-related variation in body size, hormonal status, and thermoregulation further limits extrapolation to women (Wickham et al. 2021). We retrospectively screened the EEG for overt signs of drowsiness, and the main spectral findings persisted after identified epochs were excluded. Nevertheless, without an independent measure of vigilance or sleepiness, subtle changes in arousal cannot be ruled out as a contributor to the increased delta and reduced beta/gamma power. All conditions involved a 45-min seated exposure while participants wore the same highly insulating protective ensemble (3.2 clo). The results therefore describe differences between ambient temperatures **under this clothing condition**; they cannot isolate the effect of the clothing or establish a temperature-by-clothing interaction. The ensemble’s evaporative resistance was not measured, and the seated protocol does not reproduce the physical workload of firefighters, military personnel, or hazardous-material responders. Moreover, no cognitive-performance endpoint was collected, so the EEG differences cannot be taken as evidence of impaired information processing or operational performance. Methodological limitations also apply. The spatial precision of dSPM estimates was constrained by the 32-channel montage and template anatomy (Michel and Brunet 2019), and the analysis was restricted to cortical source power. Regional wPLI was calculated between scalp electrodes; although it reduces sensitivity to zero-lag signal mixing, it cannot identify the underlying cortical connections or characterize amplitude-based coupling (He et al. 2019). Relative band power also depends on total spectral power, and conventional band measures do not separate oscillatory activity from the aperiodic spectral component (Donoghue et al. 2020). Future studies should use larger and more diverse cohorts, independent vigilance and cognitive measures, and individualized high-density source imaging. Source-space phase and amplitude connectivity, graph-theoretical and information-theoretic analyses, and time-resolved modeling could then test network integration and possible cortical–subcortical involvement (Annen et al. 2021; Panda et al. 2022, 2023). Replication and individual-level validation would be necessary before considering these EEG measures for heat-risk monitoring.

In summary, this exploratory study found temperature-associated differences across complementary EEG measures during sustained exposure while participants wore protective clothing. The 38 °C condition showed a shift toward greater low-frequency spectral power and altered regional connectivity, whereas microstate D coverage and occurrence peaked at 30 °C. These measures describe distinct aspects of ongoing EEG activity and suggest that their responses to ambient temperature need not follow the same trajectory. The study did not assess cognitive performance or establish the mechanisms underlying these differences. Whether these EEG measures can contribute to heat-risk monitoring or adaptive environmental control requires replication in larger, more diverse cohorts, individual-level predictive testing, and validation against behavioral and clinically meaningful physiological outcomes.

## Conclusion

This study examined physiological, subjective, and EEG responses to three ambient temperatures in 15 healthy young men wearing protective clothing. Mean skin temperature, core temperature, and thermal sensation increased across conditions. Higher temperatures were associated with greater relative delta power and differences in regional delta-band wPLI, while microstate D coverage and occurrence peaked at 30 °C. Source analysis also showed temperature-associated differences in estimated cortical power distribution. These findings indicate that spectral power, microstate dynamics, phase-lagged connectivity, and estimated source power capture complementary aspects of ongoing EEG activity and do not necessarily follow the same temperature trajectory. The results remain exploratory. Sensor-level connectivity and template-based source estimates cannot establish specific neural circuits, and the absence of independent vigilance and cognitive-performance measures limits functional interpretation. Larger, more diverse studies with behavioral validation are needed to determine the mechanisms and practical relevance of these EEG changes.

## Supplementary Information

Below is the link to the electronic supplementary material.

Supplementary file1 (DOCX 637 kb)

## Data Availability

Data supporting the findings of this study are available from the corresponding author upon reasonable request.

## Declarations

### Artificial Intelligence

The authors used Codex (OpenAI) to assist with the following tasks during manuscript preparation: Python scripting for statistical analysis and figure generation, editing and revision of manuscript text for clarity and concision, and formatting of the reference list. All analytical decisions, interpretation of results, and final manuscript content were made by and remain the responsibility of the authors. The use of this tool was reviewed and approved by all co-authors.
