# Supplementary material for: Acute Whole-Body Heat Exposure Modulates EEG Microstate Dynamics, Phase-Lagged Connectivity, and Cortical Source Activity
Source: Brain Topogr. 2026 Oct 7;39(6):100. doi: 10.1007/s10548-026-01258-8 (PMC13642436; doi:10.1007/s10548-026-01258-8)
Supplement: Supplementary file 1 [file 10548_2026_1258_MOESM1_ESM.docx]

**Appendix A: Supplementary source-power maps**

Supplementary Fig. A1 presents vertex-wise cortical surface maps of relative source power in the alpha, beta, gamma and theta bands under the three ambient-temperature conditions. Source power was estimated using the same inverse-model and visualization procedures as those used for the delta-band maps in Fig. 12. Within each frequency band, the same color scale was applied to all three temperature conditions to facilitate visual comparison. Color scales may differ between frequency bands.

**Fig. A1. Vertex-wise cortical distribution of relative source power across frequency bands and ambient temperatures.** Maps display the group mean relative source power on the cortical surface. A common color scale is used across temperatures within each band.
